# The effects of the age of male early life circumcision on sexual functions later in life

**DOI:** 10.1192/j.eurpsy.2021.1456

**Published:** 2021-08-13

**Authors:** E.C. Esen, S. Özer, Ö. Yıldırım, E. Hasırcı, C. Şah, B. Şahin, B. Duran, Ö. Çınar, A. Cihan, İ. Kazaz, Ü. Gül, H. Deliktaş, Y. Kızılkan, A. Altınkol, H. Kurt, Ç. Tosun, A. Güdeloğlu, İ. Üre, A. Tutuş, O. Alkış, O. Bozkurt, T. Turunç, K. Akkuş

**Affiliations:** 1 Psychiatry Department, Balikesir University, Balikesir, Turkey; 2 Urology, Dokuz Eylül Univeristy, İzmir, Turkey; 3 Urology, Cerrahpaşa University, İstanbul, Turkey; 4 Urology, Başkent University, Ankara, Turkey; 5 Urology, Özel Medline Adana Hastanesi, Adana, Turkey; 6 Urology, Marmara University, İstanbul, Turkey; 7 Urology, Samsun Eğitim ve Araştırma Hastanesi, Samsun, Turkey; 8 Urology, Zonguldak Bülent Ecevit University, Zonguldak, Turkey; 9 Urology, Niğde Eğitim ve Araştırma Hastanesi, Niğde, Turkey; 10 Urology, Karadeniz Technical University, Trabzon, Turkey; 11 Urology, Başkent University Adana Hospital, Adana, Turkey; 12 Urology, Muğla Sıtkı Koçman University, Muğla, Turkey; 13 Urology, Ankara Şehir Hastanesi, Ankara, Turkey; 14 Urology, Adana Şehir Hastanesi, Adana, Turkey; 15 Urology, Çanakkale Onsekiz Mart University, Çanakkale, Turkey; 16 Urology, Haydarpaşa Numune Eğitim ve Araştırma Hastanesi, İstanbul, Turkey; 17 Urology, Hacettepe Univeristy, Ankara, Turkey; 18 Urology, Eskişehir Osmangazi University, Eskişehir, Turkey; 19 Urology, Adıyaman University, Adıyaman, Turkey; 20 Urology, Kütahya University of Health Sciences, Kütahya, Turkey; 21 Urology, Dokuz Eylül University, İzmir, Turkey

**Keywords:** Circumcision, Premature Ejaculation, Sexual Dysfunction

## Abstract

**Introduction:**

According to psychoanalytic theory performing circumcision on a boy in phallic phase may aggravate this fear and cause sexual dysfunctions later in life. However this hypothesis is an unverified common-view rather than a scientifically proven conclusion.

**Objectives:**

We hypothesized that being circumcised during phallic phase is not a risk factor for sexual dysfunction. We also took a peak at how the experience of circumcision is being perceived and its psychological effects. Our secondary hypothesis was, sexual dysfunctions are more frequent among men who had a traumatic circumcision experience.

**Methods:**

For this cross-sectional study, a total of 2768 sexually active, circumcised and voluntary men were recruited from 20 different urology outpatient clinics around Turkey.

**Results:**

There was no significant difference for PEDT and IIEF scores between participants who were circumcised at different ages (Graph-1,2). When participants were divided into 3 groups according to their circumcision age in accordance with psychoanalytic theory (before, after and during phallic phase) mean IIEF and PEDT scores did not differ. PEDT scores did not differ either by which emotion the participant describe their experience of circumcision or how vividly he remembered it. However participants who remembered their circumcision experience more vividly and had who describe their circumcision experience with fear/anxiety had a higher IIEF score (Graph-3).

**Figure:**
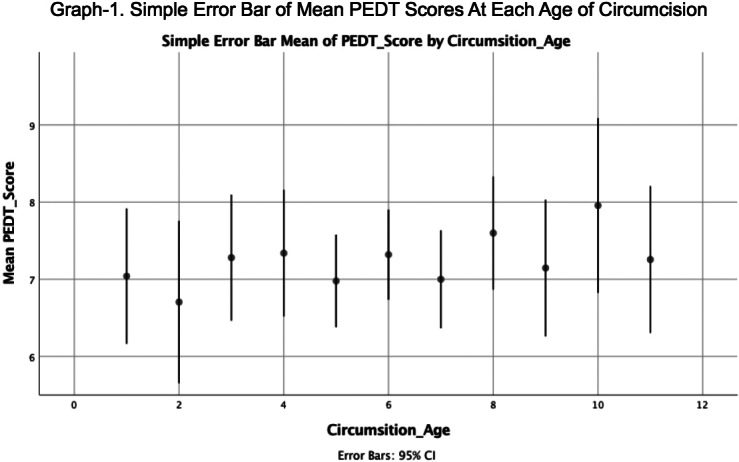

**Figure:**
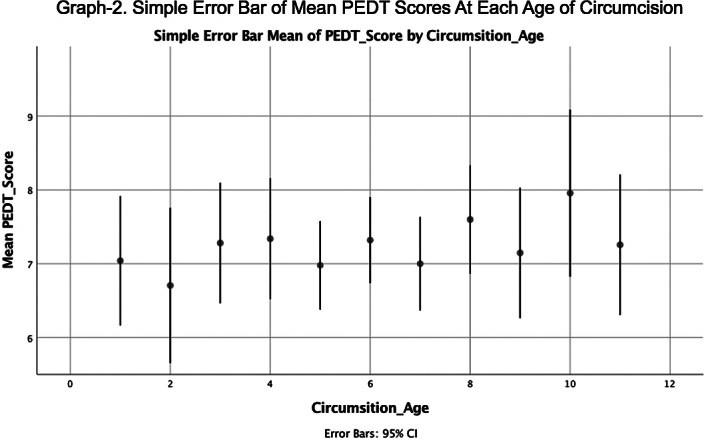

**Figure:**
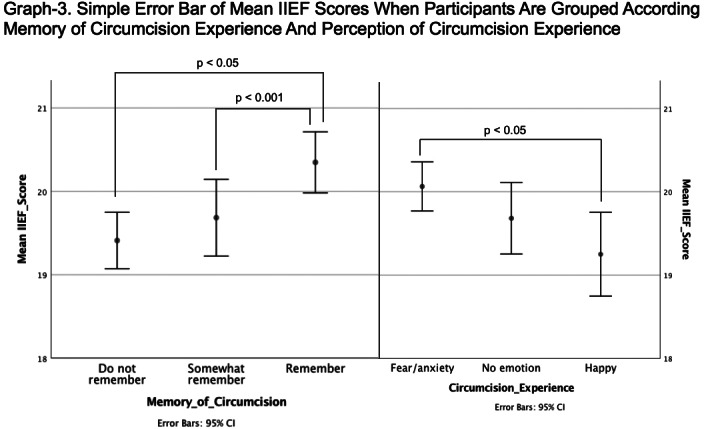

**Conclusions:**

The age of circumcision does not affect the risk of PE. This is one of the very few studies that challenges psychoanalytic theory with a scientific method. Remembering the circumcision experience with fear or anxiety did not increase the risk of sexual dysfunctions.

